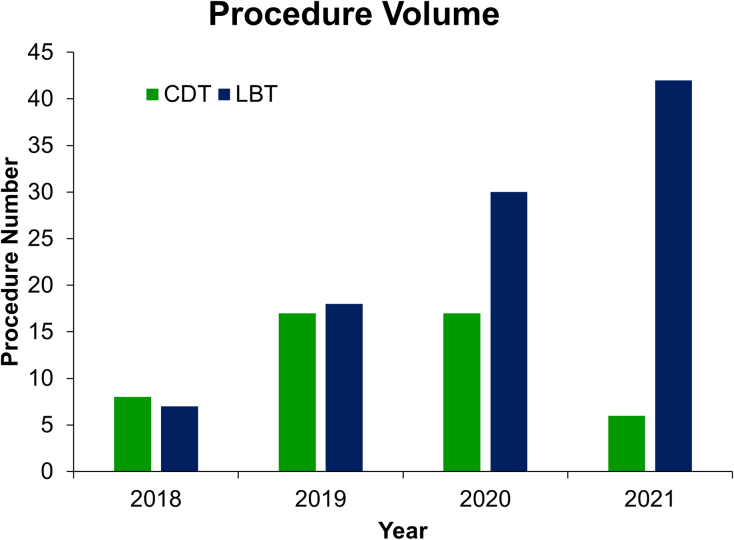# Comparison of Large-Bore Thrombectomy With Catheter-Directed Thrombolysis for the Treatment of Pulmonary Embolism

**DOI:** 10.1016/j.jscai.2022.100453

**Published:** 2023-01-04

**Authors:** Rafey Feroze, Shilpkumar Arora, Nour Tashtish, Tony Dong, Rahul Jaswaney, Yulanka Castro-Dominguez, Tarek Hammad, Mohammad Najeeb Osman, Teresa Carman, Robert Schilz, Mehdi H. Shishehbor, Jun Li

**Affiliations:** a Division of Cardiology, Department of Medicine, University Hospitals, Cleveland, Ohio; b Department of Medicine, University Hospitals, Cleveland, Ohio; c Division of Pulmonary and Critical Care Medicine, Department of Medicine, University Hospitals, Cleveland, Ohio

**Keywords:** catheter-directed thrombolysis, large-bore thrombectomy, pulmonary embolism

## Abstract

**Background:**

There is significant debate on whether large-bore thrombectomy (LBT) or catheter-directed thrombolysis (CDT) is superior for the treatment of intermediate- and high-risk pulmonary embolism (PE) while employing an early invasive strategy through endovascular therapies.

**Methods:**

Between 2018 and 2021, 147 patients who presented to our institution with acute intermediate- or high-risk PE and had undergone PE Response Team-guided endovascular intervention with either LBT (Inari FlowTriever) or CDT (EKOSonic) were retrospectively reviewed. Data on the patients' clinical characteristics, comorbidities, serum biomarkers, hemodynamics, and imaging characteristics were obtained. The primary outcome was all-cause mortality; the secondary outcomes were all-cause readmission, readmission for PE, and length of stay in the intensive care unit and hospital. The safety outcome of procedure-related bleeding was evaluated. Kaplan-Meier curves were used to estimate the cumulative event rate. Multivariate Cox-proportional hazard regression and inverse propensity weighting were used to adjust for confounders.

**Results:**

The median age of the patients was 63 (IQR, 53-73) years, and 48.3% of the patients were women. Patients in the LBT group had a higher PE Severity Index score (LBT vs CDT: median, 132 vs 108; *P* = .015) and greater prevalence of malignancy (LBT vs CDT: median, 22.7% vs 6%; *P* = .011). After propensity matching for baseline characteristics, there was no significant difference in all-cause mortality (LBT vs CDT: median, 15.8% vs 9.1%; hazard ratio, 0.64; 95% CI, 0.21-1.98; *P* = .442) for up to 1 year. The secondary outcomes or safety end points were also similar between the 2 interventions. An exploratory analysis showed elevated PE Severity Index scores, lower systolic blood pressures, and higher lactic acid levels to be associated with an increased risk of early death at 30 days.

**Conclusions:**

In this retrospective cohort study, there was no significant difference in the cumulative event rate of all-cause mortality between LBT and CDT. Further studies are needed to evaluate the use of LBT versus CDT versus noninvasive therapy to understand outcomes and appropriate patient selection among those with intermediate- and high-risk PE.

## Highlights

• Catheter-directed thrombolysis and large-bore thrombectomy can be used to treat pulmonary embolism.
• The mortality and rehospitalization rate were similar between both the approaches.
• The safety profiles of both the approaches were similar.
• Patients who died within the first 30 days had worse clinical markers.

## Central Illustration

**Figure:**
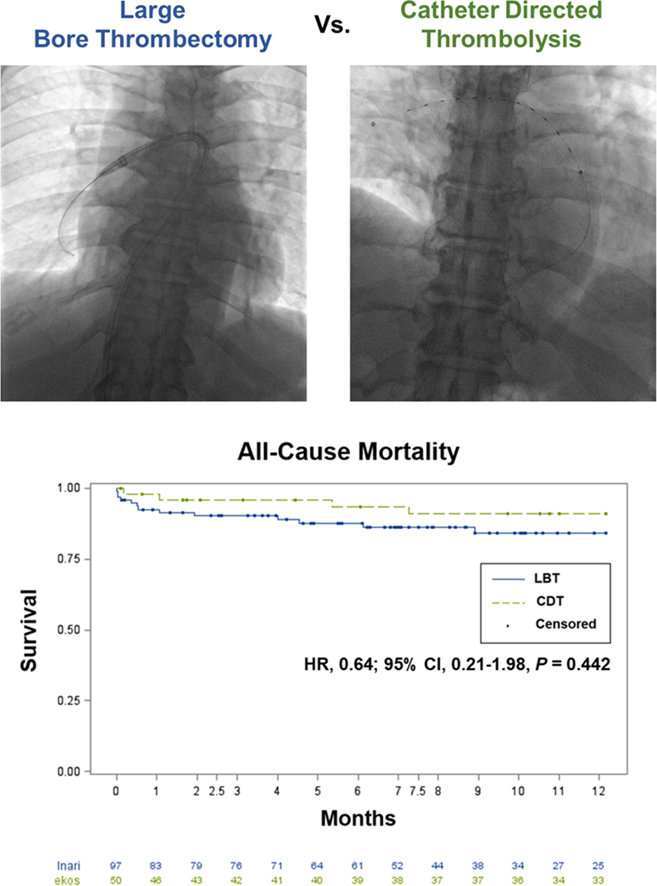

## Introduction

Pulmonary embolism (PE) constitutes a significant burden of disease worldwide. In the United States alone, PE is estimated to result in 100,000 deaths per year, rendering it the third leading cause of death due to cardiovascular disease.^1^^,^^2^ In addition, PE has also been identified as one of the major complications of the now ubiquitous COVID-19, with an associated increase in mortality rate of 45% among patients with COVID-19 who develop PE compared with that in those without PE.^3^

Risk stratification of patients with PE depends on noninvasive hemodynamics, imaging findings, biomarkers suggestive of right ventricular strain, and antecedent comorbidities.^4^, ^5^, ^6^, ^7^ Based on these characteristics, patients are categorized as having a low, submassive or intermediate, or massive or high risk.^2^ PE Response Teams (PERTs) have been implemented across many countries to help with rapid decision making to determine the best route of treatment based on the severity of obstructive shock and hemodynamic compromise.^8^ The treatment options include systemic anticoagulation, systemic thrombolysis, endovascular treatment, and surgical embolectomy.^9^ However, variations in society guidelines and limited trial data make treatment selection challenging. The choice of treatment is often dictated by the degree of hemodynamic compromise, thrombotic burden, anatomy and comorbidities, bleeding assessment, and operator or institutional experience.

In the last decade, the advent of catheter-directed thrombolysis (CDT) and large-bore thrombectomy (LBT) has changed the landscape of endovascular treatment options for patients with intermediate- and high-risk PE.^10^, ^11^, ^12^, ^13^, ^14^, ^15^ Similar to some other institutions across the country, our system has transitioned from a CDT-primary treatment modality to a LBT-primary treatment modality in the last 3 years. However, no head-to-head comparison is currently available to assess the clinical outcomes of these treatment options. In this study, we sought to compare our system's outcomes in patients who underwent LBT with those in patients who underwent CDT for intermediate- and high-risk PE.

## Materials and methods

### Patient population

The health care system of University Hospitals comprises 11 hospitals across northeast Ohio, with a robust systemwide PERT program. We evaluated our patients who received endovascular intervention with either LBT using the Inari FlowTriever thrombectomy catheter (Inari Medical) or CDT using the EKOSonic device (Boston Scientific) between February 2018 and August 2021. The decision to pursue mechanical intervention for PE was made by a multidisciplinary team consisting of a primary team provider, pulmonologist, interventional cardiologist, and vascular medicine physician. The criteria for endovascular intervention were based on a variety of clinical and patient-specific markers, including but not limited to the following: (1) PE identified using the computed tomography chest PE (CTPE) protocol; (2) presence of right ventricular strain detected using the CTPE protocol and/or transthoracic echocardiography; (3) elevated levels of cardiac and noncardiac biomarkers (eg, troponin I, brain natriuretic peptide [BNP] and lactic acid); (4) hemodynamic compromise evidenced by blood pressure and/or heart rate derangements; and (5) persistently abnormal oxygen saturations. Those with aortic dissection; prior intracranial hemorrhage; known intracranial infarcts, masses, or neoplasm; ischemic stroke within 3 months; recent surgery; or other absolute contraindications to thrombolysis were precluded from CDT-based treatment. The choice of LBT versus CDT among remaining patients was based on operator preference; this was typically dependent on index hemodynamics, thrombotic burden and location, and feasibility of one modality versus another in the specific hospital branch. Following endovascular PE intervention, all patients received postprocedure care in the intensive care unit (ICU), with transition to an individualized anticoagulation strategy based on the glomerular filtration rate, presence of mechanical valve, malignancy, and/or insurance coverage at discharge.

### Baseline variables

Patient demographics (eg, age, sex, and race), comorbidities (eg, hypertension, hyperlipidemia, heart failure, chronic obstructive pulmonary disease, malignancy, and smoking history), preprocedure vital signs (heart rate, blood pressure, respiratory rate, and oxygen saturation), preintervention invasive hemodynamics (right atrial and pulmonary artery pressures, cardiac output, and cardiac index), and prehospitalization medications were obtained from chart review (Table 1). The PE Severity Index (PESI) score was calculated using the abovementioned variables.^16^ The ratio of the diameter of the right ventricle (RV) to that of the left ventricle (LV) was obtained by measuring the width of the ventricular cavities immediately adjacent to the tricuspid and mitral valves and perpendicular to the long axis of the LV on axial images of the preintervention CTPE protocol, optimized to represent a coaxial 4-chamber view of the heart.

**Table 1:** Baseline characteristics of patients.

Baseline characteristics	Overall patients	LBT	CDT	*P* value
Total number of patients, n	147	97	50	
Age	63 (52-73)	65 (53-74)	61 (48-68)	.149
Female	71 (48.3%)	44 (45.4%)	27 (54%)	.321
White	109 (74.2%)	73 (75.3%)	36 (72%)	
Serum biomarkers				
Troponin I, ng/mL	0.30 (0.09-1.00)	0.39 (0.11-1.08)	0.25 (0.40-0.73)	.084
Brain natriuretic peptide, pg/mL	110 (41-361)	148.5 (51.5-356.5)	78 (28-361)	.109
Lactic acid, mmol/L	2.0 (1.4-3.2)	2.0 (1.4-3.4)	1.9 (1.5-3.0)	.663
PESI score	121 (95-171)	132 (102-177)	108 (86-140)	.015∗
RV/LV ratio	1.34 (1.23-1.69)	1.36 (1.01-1.83)	1.29 (1.13-1.55)	.24
Comorbidities				
Dyslipidemia	82 (55.8%)	56 (57.7%)	26 (52%)	.507
Hypertension	107 (72.2%)	72 (74.2%)	35 (70%)	.585
Coronary artery disease	145 (98.6%)	96 (99%)	49 (98%)	.631
Heart failure	46 (31.3%)	33 (34.0%)	13 (26%)	.32
Chronic obstructive pulmonary disease	42 (28.6%)	24 (24.7%)	18 (36.0%)	.152
Malignancy	25 (17%)	22 (22.7%)	3 (6%)	.011∗
Smoking	82 (53.6%)	52 (53.6%)	30 (60%)	.46
Vital signs				
Systolic blood pressure, mm Hg	100 (88-117)	96 (85-114)	107 (96-121)	.026∗
Heart rate, beats/min	112 (101-125)	112 (100-125)	112 (103-124)	.773
Respiratory rate, breaths/min	24 (20-30)	24 (22-30)	24 (20-29)	.218
Temperature, °F	97.1 (96.6-97.7)	97.1 (96.6-97.7)	97.3 (96.6-97.7)	.218
Oxygen saturation, %	90 (85-93)	90 (84.5-93.0)	90.0 (87-94.0)	.432
Preintervention invasive hemodynamics				
Number of patients with PA pressure measurements, n	99	67	32	
RA mean pressure, mm Hg	13 (8-16)	13 (8-17)	10 (8-15)	.454
PA systolic pressure, mm Hg	52 (43-63)	49 (43-61)	57 (48-64)	.088
PA diastolic pressure, mm Hg	20 (12-24)	18 (11-24)	20 (16-23)	.628
PA mean pressure, mm Hg	32 (26-38)	32 (25-37)	35 (27-39)	.312
Number of patients with Fick CO or CI measurements, n	57	28	29	
PA saturation, %	60 (56-68)	59 (51-67)	61 (57-69)	.052
Cardiac output, L/min	4.29 (4.03-5.43)	4.56 (4.12-5.85)	4.22 (3.77-5.16)	.906
Cardiac index, L/min/m^2^	2.15 (1.88-2.65)	2.30 (1.90-2.90)	2.10 (1.80-2.45)	.655
Anticoagulation at discharge				
Direct oral anticoagulant	99 (67.3%)	61 (62.8%)	38 (76.0%)	.108
Vitamin K antagonist	35 (23.8%)	24 (24.7%)	11 (22.0%)	.712

Continuous variables are presented as median and interquartile range. Categorical variables are presented as absolute number (%). Pulmonary artery saturation was available for 39% of the entire cohort. Cardiac output and cardiac index were available for 31% of the patients. A small number of patients (n = 13) did not receive anticoagulation at discharge either because of in-hospital death or absolute contraindications to anticoagulation.

CDT, catheter-directed thrombolysis; CI, cardiac index; CO, cardiac output; LBT, large-bore thrombectomy; LV, left ventricle; PA, pulmonary artery; PESI, Pulmonary Embolism Severity Index; RA, right atrial; RV, right ventricle.

∗ *P* < .05.

### Outcomes

The primary outcome was all-cause mortality up to 1 year. We utilized a social security database to verify mortality and date of death. The secondary outcomes were all-cause readmission up to 1 year, readmission because of PE up to 1 year, index hospitalization length of stay, and ICU length of stay. The secondary safety outcome was analyzed for major bleeding as defined by the development of intracranial hemorrhage, a hemoglobin drop of ≥3 g/dL, and/or need for blood transfusion. The bleeding outcomes were queried within a 7-day period to capture procedure-related complications.

### Statistical analysis

Continuous variables are presented as median with IQR. Categorical variables are presented as total numbers and percentages. Continuous variables (nonparametric) were compared using the Kruskal-Wallis test, and categorical variables were compared using the χ^2^ test. Kaplan-Meier curves were generated to estimate the cumulative event rate.

Multivariate Cox-proportional hazard regression was utilized to adjust for confounders. Previous literature was used to identify variables (age, sex, RV/LV ratio, PESI score, troponin, BNP, and lactic acid) that impact mortality,^17^, ^18^, ^19^, ^20^ and these were then adjusted for in a multivariate model. Furthermore, a sensitivity analysis was conducted using inverse probability (propensity) of the treatment weighting method. A propensity score, using the same variables as those in the Cox-proportional hazard regression, was generated using a multivariable logistic regression model. Subsequently, a double-robust method was used to generate treatment weights (inverse of propensity) and adjusted with propensity for robust matching.^21^

For all the statistical tests, a *P* value of <.05 was indicative of statistical significance. All the statistical analyses were performed using the IBM SPSS, version 26, and SAS, version 9.4, analytics software. This study was approved by the local institutional review board, with a waiver of consent. Clinical data were collected from the patients' electronic medical records and entered into the Research Electronic Data Capture tool hosted at University Hospitals Cleveland Medical Center.^22^

## Results

### Patient population

Between February 2018 and August 2021, a total of 147 patients who presented with symptoms of submassive or massive PE underwent endovascular intervention with either LBT (n = 97) or CDT (n = 50) (Table 1). The median age of the patients in our cohort was 63 (IQR, 52-73) years, and 48.3% were women. The most common comorbidities were hypertension (72.2%), dyslipidemia (55.8%), and active smoking (53.6%). The median heart rate was 112 (IQR, 101-125) beats/min, median systolic blood pressure (SBP) was 100 (IQR, 88-117) mm Hg, and median oxygen saturation was 90% (IQR, 85%-93%). Troponin I and BNP levels were minimally elevated and similar in both the groups. All the baseline characteristics were similar between the 2 intervention groups, except higher PESI scores (LBT vs CDT: median, 132 vs 108; *P* = .015) and higher rates of malignancy (LBT vs CDT: 22.7% vs 6%; *P* = .011) in the LBT cohort.

### Primary outcome

Our hospital system's practice predominately featured the use of CDT in 2018, with the use of LBT becoming more frequent in 2019 and, thereafter, with an associated decrease in CDT cases (Supplemental Figure S1). The median follow-up duration was 284 days. There was no significant difference in all-cause mortality at 1 year between the LBT and CDT cohorts (LBT, 15.8%; CDT, 9.1%). Univariate Cox regression (hazard ratio [HR], 0.54; 95% CI, 0.17-1.62; *P* = .264) and multivariate Cox-proportional regression hazard models (HR, 0.64; 95% CI, 0.21-1.98; *P* = .442) did not demonstrate a significant difference in mortality with LBT compared with that with CDT. Inverse propensity weighting also did not demonstrate a significant difference in mortality with LBT compared with that with CDT (HR, 0.57; 95% CI, 0.27-1.18; *P* = .128) (Table 2). Kaplan-Meier curves for mortality at 1 year demonstrated early but statistically insignificant separation (Central Illustration).

**Table 2:** Primary and secondary outcomes.

Outcomes	Overall	LBT	CDT	*P* value
Total number of patients, n	147	97	50	
All-cause mortality, n	17	13	4	
Cumulative estimates rate, %				
At 30 d	5.6	7.4	2.1	
At 3 mo	7.8	9.7	4.2	
At 1 y	13.4	15.8	9.1	
Univariate, HR (95% CI)		0.53 (0.17-1.62)		.264
Multivariate Cox regression, HR (95% CI)		0.64 (0.21-1.98)		.442
Inverse propensity weighting, HR (95% CI)		0.57 (0.27-1.18)		.128
All-cause readmission, n	42	29	13	
Cumulative estimates rate, %				
At 30 d	10.5	11.4	8.8	
At 3 mo	23.3	28.2	13.4	
At 1 y	37.7	39.3	33	
Univariate, HR (95% CI)		0.71 (0.37-1.38)		.314
Multivariate Cox regression, HR (95% CI)		0.79 (0.40-1.53)		.477
Inverse propensity weighting, HR (95% C])		0.78 (0.51-1.20)		.25
Readmission for pulmonary embolism, n	5	3	2	
Cumulative estimates, %				
At 30 d	1.5	1.2	2.1	
At 3 mo	3.2	3.8	2.1	
At 1 y	4.8	3.8	4.9	
Univariate, HR (95% CI)		1.04 (0.17-6.44)		.967
Multivariate Cox regression, HR (95% CI)		0.92 (0.14-5.84)		.927
Inverse propensity weighting, HR (95% CI)		0.73 (0.19-2.82)		.649
Length of stay in hospital, d, median (IQR)	6 (4-10)	6 (4-10)	6 (4-10)	.355
Length of stay in ICU, d, median (IQR)	3 (2-5)	3 (2-5)	3 (2-5)	.97
Postprocedural safety outcome (within 7 d of procedure)				
Cumulative major complications	23/142 (16.2%)	18/92 (19.6%)	6/50 (12%)	.139
Intracranial hemorrhage	1/142 (0.7%)	1/92 (1.1%)	0/50 (0%)	—
Hemoglobin drop ≥ 3 g/dL	21/142 (14.8%)	16/92 (17.4%)	5/50 (10%)	.236
Required blood transfusion	2/142 (2.1%)	2/92 (2.1%)	1/50 (2%)	.396

Cumulative event rates were calculated using Kaplan-Meier estimates.

CDT, catheter-directed thrombolysis; HR, hazard ratio; ICU, intensive care unit; LBT, large-bore thrombectomy.

**Central Illustration:**
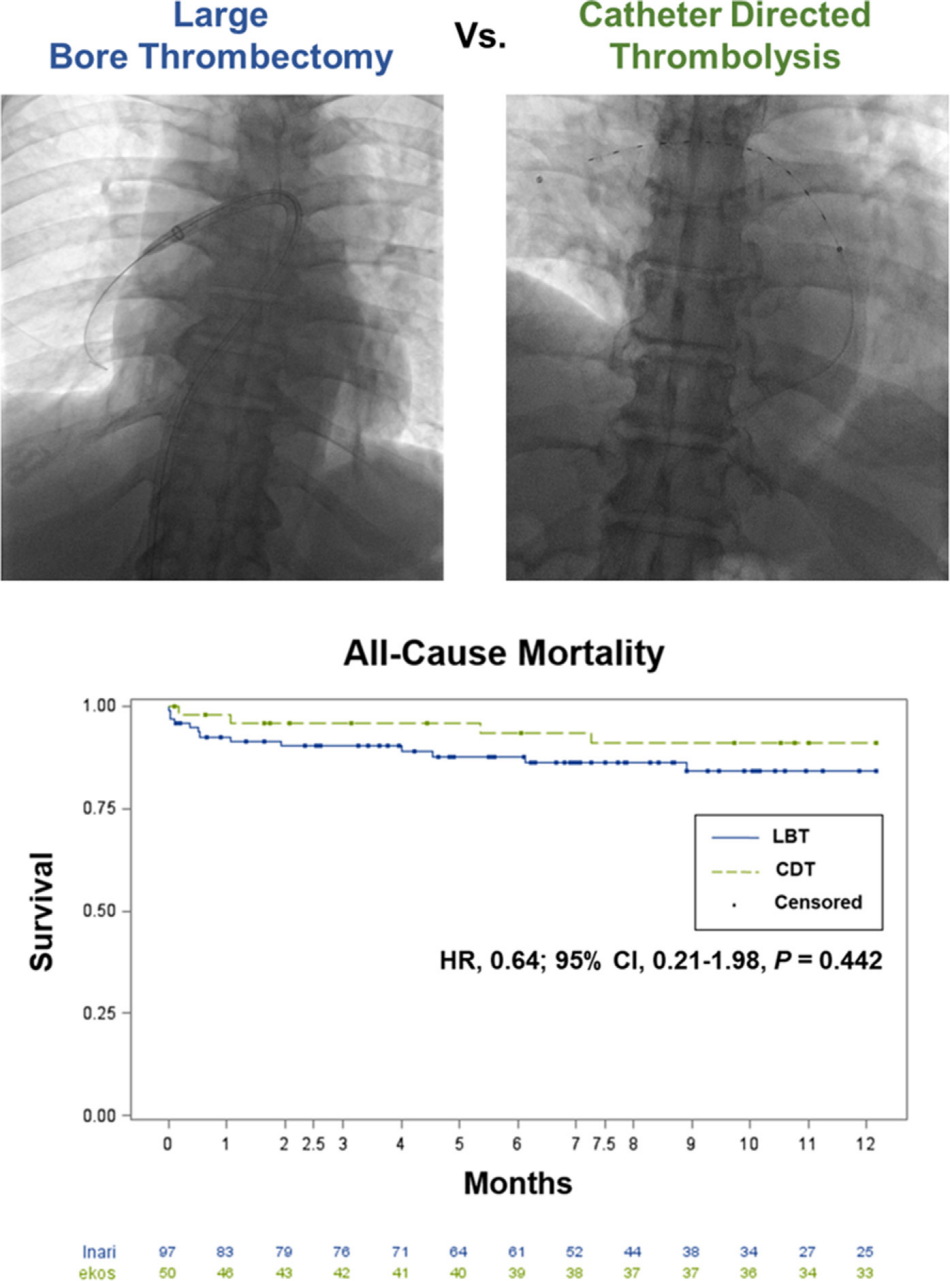
**Representative fluoroscopic images demonstrating large-bore thrombectomy (LBT) (top left) and catheter-directed thrombolysis (CDT) (top right) with unadjusted Kaplan-Meier curves (bottom half)comparing all-cause mortality between LBT and CDT.** HR, hazard ratio.

### Secondary outcomes

**Figure 1:**
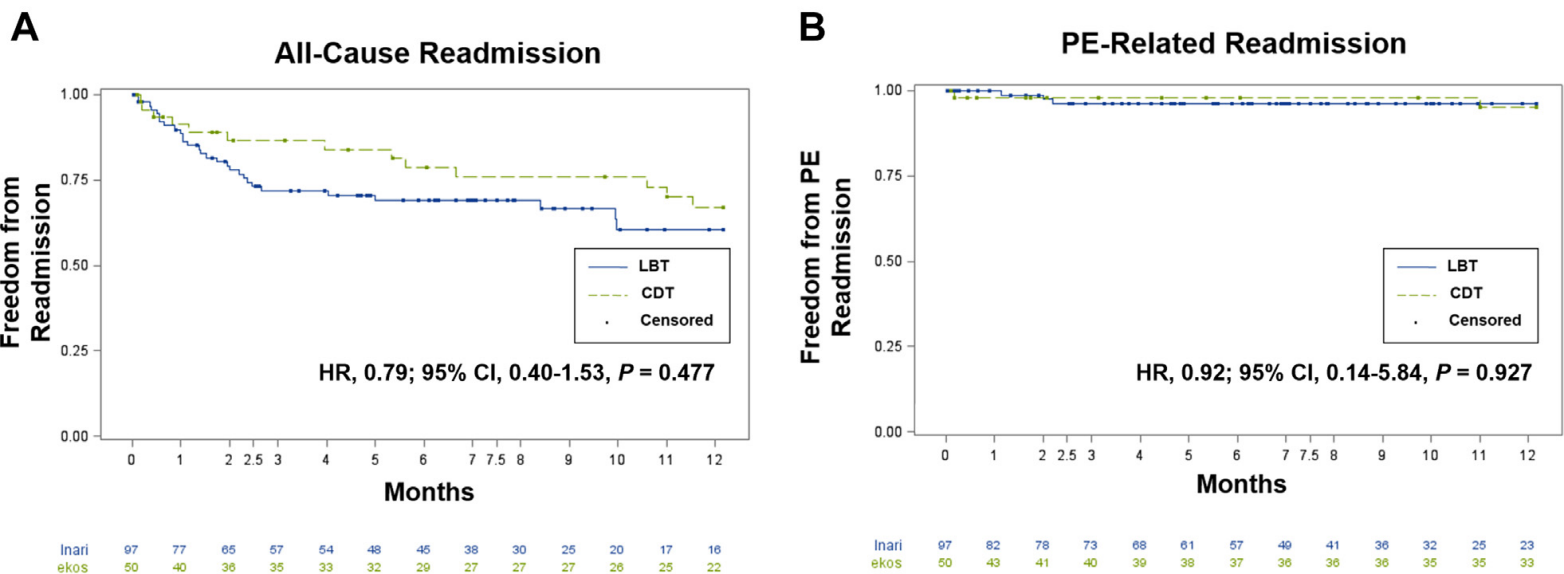
**Unadjusted Kaplan-Meier curves comparing (A) all-cause readmission and (B) pulmonary embolism (PE)-related readmission between large-bore thrombectomy (LBT) and catheter-directed thrombolysis (CDT).** HR, hazard ratio.

All-cause rehospitalization (LBT vs CDT: 39.3% vs 33.0%; HR, 0.79; 95% CI, 0.40-1.53; *P* = .477) (Figure 1A) and readmission for PE were not statistically significantly different (LBT vs CDT: 3.8% vs 4.9%; HR, 0.92; 95% CI, 0.14-2.82; *P* = .649) (Figure 1B) for the 2 treatment groups. The median lengths of ICU and hospital stay were also not significantly different (Table 2).

The secondary outcomes pertaining to the safety bleeding profile between LBT and CDT did not reveal significant differences. We did not have bleeding data on 5 patients. Of the remaining patients, 1 was found to have intracranial bleeding following failed LBT with augmentative systemic tissue plasminogen activator (tPA) and emergency venoarterial extracorporeal membrane oxygenation (VA-ECMO) initiation, which was complicated by ischemic stroke and hemorrhagic conversion (Table 3). There was no significant difference in the incidence of a hemoglobin drop of ≥3 g/dL (LBT vs CDT: 17.4% vs 10.0%; *P* = .236) or the requirement for blood transfusion (LBT vs CDT: 3.2% vs 2%; *P* = .665) within 7 days of the procedure. The cumulative composite of these safety outcomes, while being numerically higher for the LBT group (LBT vs CDT: 19.6% vs 12%; *P* = .139), did not reach statistical significance.

**Table 3:** Patient-level analysis of cause of death within 1 year of intervention.

Age (years)	Sex	PESI	Intervention	Days from procedure to mortality	Presenting symptom arrest	VA-ECMO	Cause of death
71	M	231	LBT	0	Yes	No	Underlying Parkinson and wheelchair bound, deteriorating clinically in the prior year. Moderate amount of thrombus retrieved. Escalating pressors, PEA after procedure, without ROSC.
58	F	228	LBT	1	Yes	Pre	OHT 3 mo prior complicated by RV failure, requiring Protek Duo × 1 month. Presented with arrest, VA-ECMO initiated after 1 h of CPR. Minimal thrombus removal. Escalating pressor support, transitioned to CC.
75	F	215	LBT	1	No	No	Minimal thrombus removal. Not a candidate for VA-ECMO because of age and comorbidities. Escalating pressor support, transitioned to CC.
67	F	248	LBT	3	No	Intraprocedural	COVID-19-related PE. Intraprocedural arrest requiring VA-ECMO. Minimal thrombus removal; intraprocedural tPA at 50 mg, with improved perfusion. Complicated by ischemic stroke and subsequent hemorrhagic conversion, transitioned to CC.
44	F	135	CDT	5	No	Intraprocedural	Prior complex uterine surgery, with submassive PE and DVT. Initially, LBT with minimal thrombus removal. Vasovagal episode 1 d later. Taken back for CDT because of concerns of LBT failure, PEA on arrival, emergently placed VA-ECMO. CDT performed for 2 h, stopped because of hemorrhagic tamponade, requiring pericardiocentesis. Multiorgan failure, transitioned to CC.
59	F	149	LBT	11	No	No	Stage IV-B NSCLC with diffuse metastasis and prognosis of 6-mo life expectancy. Progressive hypoxia and hypotension, with good thrombus extraction. Continued to have clinical deterioration owing to NSCLC, transitioned to CC.
77	M	177	LBT	15	No	No	Underlying vascular dementia, admitted with AMS and SAH, and managed conservatively. Developed syncope and hypoxia while inpatient. Found to have bilateral PE and underwent LBT with good thrombus removal and IVC filter placement. De-escalated from ICU and discharged but readmitted soon thereafter, with DKA and transitioned to CC.
83	F	163	LBT	16	No	No	Decompensated cirrhosis and hepatocellular carcinoma, with global decline over the prior year. Presented with dyspnea due to PE and underwent LBT with good thrombus extraction and discharged on Apixaban. Presented again with AMS and lethargy and not deemed to be a transplant candidate. Given global decline since malignancy diagnosis, transitioned to CC.
89	M	219	CDT	32	No	No	Chronic debility. Presented with recurrent falls, dyspnea, and hypoxia. CDT removed early because of development of groin hematoma, which required 4 units of blood transfusion, followed by surgical evacuation and IVC filter placement. Discharged AMA and lost to follow-up. Death discovered by social security database.
53	M	173	LBT	32	No	No	Presented with PE and metastatic SCLC, with occlusion of right lower and middle lobe bronchi. Good bilateral thrombus removal. Course complicated by intracranial hemorrhage due to brain metastases. IVC filter placed but found to have right atrial thrombus. Developed bacteremia and transitioned to hospice.
74	M	134	LBT	58	No	No	Prior history of type B aortic dissection, with repair presented with acute type A aortic dissection and PE. Good thrombus extraction and IVC filter placement followed by surgical repair of aortic dissection complicated by hemorrhagic shock and cardiac tamponade requiring pericardial window. Transferred to LTACH with final cause of death unknown.
34	F	204	LBT	120	No	No	History of DVT/PE and desmoplastic pelvic small round tumor with extensive metastases precluding surgical candidacy and palliative chemotherapy. Presented with fatigue, dyspnea, hypotension, tachycardia, and hypoxia after cessation of Apixaban with a history of ITP. Bilateral, upper, and lower lobe segmental and subsegmental PEs were found. Minimal clot extracted. Course complicated by chemo/immunotherapy intolerance and ITP. Developed malignant compressive pleural effusions and ascites, resulting in worsening hypoxia. Not deemed to be a candidate for further cancer therapies and transitioned to hospice.
65	M	205	LBT	136	No	No	Presented with hypoxia and lower extremity DVT with saddle PE. Significant thrombus removal along with IVC filter placement. Returned 2 mo later with gross hematuria and bladder cancer with metastases to the brain, bone, and spine discovered. Developed bilateral hydronephrosis and AMS and transitioned to CC.
41	M	81	CDT	161	No	No	Unprovoked saddle PE with bilateral extension refractory to systemic thrombolytics with improvement following CDT. Lost to follow-up until presented with PEA arrest of unknown etiology without ROSC despite systemic thrombolytics.
82	F	121	LBT	184	No	No	History of hip surgery and decreased mobility. Admitted after a fall and found to have extensive LE DVT and also had removal of clot burden from IVC, discharged to a rehabilitation facility, and lost to follow-up.
54	M	192	CDT	218	No	No	Progressive dyspnea for 1 wk and presented with presyncope and CP. Found to have bilateral PE with RV strain and LE DVT. Underwent CDT, with resolution of symptoms and discharged but lost to follow-up.
82	M	110	LBT	267	No	No	History of dementia, vascular disease, systolic heart failure, and prior PE presented with acute dyspnea and found to have bilateral PE. Underwent LBT, with good thrombus removal and resolution of symptoms. Discharged but lost to follow-up.

AMA, against medical advice; AMS, altered mental status; CC, comfort care; CDT, catheter-directed thrombolysis; CPR, cardiopulmonary resuscitation; DKA, diabetic ketoacidosis; DVT, deep vein thrombosis; ICU, intensive care unit; ITP, idiopathic thrombocytopenic purpura; IVC, inferior vena cava; LBT, large-bore thrombectomy; LE, lower extremity; LTACH, long-term acute care hospital; NSCLC, non–small cell lung cancer; OHT, orthostatic heart transplant; PE, pulmonary embolism; PEA, pulseless electrical activity; PESI, Pulmonary Embolism Severity Index; ROSC, return of spontaneous circulation; RV, right ventricle; SAH, subarachnoid hemorrhage; SCLC, squamous cell lung cancer; tPA, thrombin plasminogen activator; VA-ECMO, venoarterial extracorporeal membrane oxygenation.

### Mortality outcomes

We further reviewed patient-level data for all-cause mortality in both the arms until 1 year of follow-up (Table 3). Six patients (5 who underwent LBT and 1 who underwent CDT) died during the index hospitalization. All 6 had significant hemodynamic compromise due to RV collapse with half of them requiring emergency VA-ECMO for stabilization either before or during the procedure. Of the 5 patients who underwent LBT, only minimal thrombus retrieval was possible in 3, typically suggestive of a subacute-to-chronic presentation of PE. Two of the patients who underwent LBT died of non-PE-related causes. LBT had failed 24 hours prior, with scant thrombus retrieval, in the patient who underwent CDT and died during the index hospitalization.

In the LBT group, 2 additional patients died during the first 30 days but outside of the index hospitalization, and 4 patients died within 6 months. A patient who underwent CDT developed a groin hematoma, requiring blood transfusion and surgical evacuation, but left against medical advice and was noted to be deceased based on the social security database. An exploratory analysis of the clinical characteristics of patients who passed away during the first 30 days, compared with those of patients alive at 30 days, revealed that the former cohort had lower mean SBPs (*P* = .028), higher PESI scores (*P* < .001), and higher mean lactic acid levels (*P* = .036) (Table 4).

**Table 4:** Clinical markers associated with mortality within 30 days for all comers regardless of large-bore thrombectomy or catheter-directed thrombolysis as a treatment modality.

Clinical marker	Mortality within 30 d (n = 8)	Alive at 30 d (n = 139)	*P* value
Age, y	69.5 (58.5-76.5)	63 (51-73)	.240
PESI	196 (156-229)	112 (94-162)	**<.001**
Heart rate, beats/min	119 (105-136)	112 (100-124)	.293
Systolic blood pressure, mm Hg	77 (62-103)	101 (89-117)	**.028**
Troponin I, ng/mL	0.96 (0.28-1.36)	0.27 (0.08-0.96)	.115
BNP, pg/mL	94 (55-605)	112 (40-355)	.771
Lactic acid, mmol/L	2.5 (2.2-5.8)	1.9 (1.4-3.1)	**.036**
RV/LV ratio	1.35 (1.13-1.65)	1.31 (1.10-2.30)	.615

BNP, brain natriuretic peptide; LV, left ventricle; PESI, Pulmonary Embolism Severity Index; RV, right ventricle.

Significant *P*-values presented as bold.

## Discussion

In this retrospective cohort study of patients presenting with intermediate- and high-risk PE undergoing either LBT or CDT, there was no significant difference in the cumulative event rate of all-cause mortality for up to 1 year (Central Illustration), as determined using univariate, multivariate, and inverse propensity weighting analyses. Examination of secondary endpoints did not demonstrate significant differences in all-cause readmission, PE-specific readmission, index length of stay, or length of ICU stay between LBT and CDT.

The overall mortality rate and long-term clinical sequela associated with patients presenting with intermediate- and high-risk PE are not inconsequential^2^ and depend on thrombus burden, comorbidities, and tolerance of the RV to acute obstructive strain. CDT has historically been shown to be superior to anticoagulation alone,^10^^,^^11^^,^^15^ but primarily has the end point of improved early RV/LV ratios. The recent popularization of LBT has rendered it to be the primary modality of treatment in some institutions, although there is an ongoing debate on which of these is the better approach for patients presenting with intermediate- and high-risk PE.^23^ To our knowledge, this is the largest analysis to date comparing LBT and CDT for treatment of submassive and massive PE in a health care system.

The low rate of 30-day all-cause mortality in the CDT group was similar to what has been reported in previous trials with targeted fibrinolytic therapy. The Ultrasound Accelerated Thrombolysis of Pulmonary Embolism (ULTIMA) and Submassive and Massive Pulmonary Embolism Treatment With Ultrasound Accelerated Thrombolysis Therapy (SEATTLE II) trials, which evaluated the use of CDT with the EKOSonic catheter system, reported a 30-day mortality of 0% and 2.7%, respectively.^10^^,^^11^ Of note, the total dose of tPA in our CDT arm ranged from 12 to 24 mg and was comparable with the dosing regimen used in these studies but higher than the dosing regimens tested in the recent Optimum Duration of Acoustic Pulse Thrombolysis Procedure in Acute Pulmonary Embolism (OPTALYSE) trial,^15^ suggesting that a lower dose of tPA can be sufficient in reducing RV strain in such patients. Further study of the comparison of lower-dosage regimens for CDT versus those for LBT in terms of mortality and the risk of bleeding is required.

The 30-day mortality in our LBT group was higher than what has been reported in prior studies of thrombectomy for the treatment of PE. Use of the Indigo Aspiration system (Penumbra) demonstrated a mortality of 2.5% at 30 days with 1 device-related death at 48 hours.^12^ However, our LBT group had a higher risk profile, with a lower mean SBP, higher heart rate, and greater proportion of patients with troponin I elevation, suggesting more hemodynamic compromise. The FlowTriever Pulmonary Embolectomy Clinical Study (FLARE) and FlowTriever All-Comer Registry for Patient Safety and Hemodynamics (FLASH) ,^13^^,^^14^ both featuring the Inari FlowTriever system, reported a 30-day mortality of 1% and 0.4%, respectively. However, the FLARE trial did not include any patients with a simplified Pulmonary Embolism Severity Index (sPESI) score of >1, and the mean sPESI score in the FLASH registry was reported to be 1.6. Comparatively, our LBT group had a mean sPESI score of 2.3, representing a more real-world patient cohort with higher hemodynamic compromise because of PE.

We noted early but statistically insignificant separation of Kaplan-Meier curves for all-cause mortality, readmission for PE, and readmission for any cause. However, the patients in the LBT cohort had significantly higher PESI scores than those who underwent CDT. Given that the Kaplan-Meier curves were unadjusted for clinical factors, this may be reflective of a higher-risk patient population that is less tolerant of hemodynamic compromise and more prone to readmission for greater comorbid conditions. Indeed, our patient-level analysis of mortality during the index hospitalization showed that 50% of the patients with in-hospital mortality required VA-ECMO placement, either periprocedurally or intraprocedurally, for full circulatory support. Patients who underwent LBT and CDT and died within 30 days had not only higher PESI but also lower SBPs and higher mean lactic acid values than those alive at 30 days, indicating a significantly sicker cohort of patients. Lastly, malignancy was represented in a higher proportion in the LBT group, potentially contributing to increased mortality rates at 6 and 12 months, along with greater readmissions, and not necessarily reflective of hemodynamic compromise because of the initial PE or procedure.

The management of patients with intermediate- and high-risk PE in the current era is a rapidly evolving field. Systemic anticoagulation remains the cornerstone of therapy for all patients with PE, with systemic tPA and surgical options for unstable, high-risk patients depending on bleeding profiles.^2^^,^^9^ Alternatively, immediate hemodynamic support with VA-ECMO and thrombectomy has been introduced as a potential alternative in appropriate patients with high-risk PE with significant hemodynamic embarrassment and/or cardiac arrest^24^; however, further evaluation is needed to assess this treatment pathway. For patients with intermediate-to-high-risk PE, in the absence of profound and rapidly deteriorating hemodynamics, endovascular therapy is a reasonable first approach. Some practices around the country have quickly adapted an LBT-primary approach in the last few years, particularly in light of the COVID-19 pandemic, a perceived shortened length of hospital stay, and the availability of multiple new LBT devices. However, we still have absence of data comparing LBT versus CDT in a head-to-head trial.

Our real-world application of LBT and CDT has shown equivalent outcomes in terms of all-cause mortality, readmission, length of stay, and safety outcomes. The early death rates, although nonsignificant, were higher in the LBT cohort; however, this finding may be correlated with higher PESI scores and more hemodynamic compromise. We look forward to the results of the currently enrolling PEERLESS randomized trial evaluating the outcomes of LBT versus CDT in patients with intermediate-to-high-risk PE.^25^ Future endeavors should evaluate the adjunctive use of early hemodynamic support via VA-ECMO to help stabilize rapid RV collapse in appropriate patients with high-risk PE and progressive obstructive shock.^24^ Lastly, although early invasive strategies via endovascular therapies have been shown to improve RV/LV ratios after treatment,^11^, ^12^, ^13^ the evaluation of long-term clinical outcomes (eg, improvement of quality of life and objective measurements of dyspnea) comparing CDT with anticoagulation alone is ongoing.^26^

Our study has limitations akin to other retrospective chart review studies. First, pretreatment transthoracic echocardiography and invasive pulmonary artery pressures were not ubiquitously available because of the emergent status of these procedures. Additionally, the 2 treatment groups had different baseline characteristics, with a greater incidence of malignancy and higher PESI score in the LBT group. To adjust for these discrepancies, multivariate analysis and inverse propensity weighting were performed to mitigate these baseline differences. Third, a matched control group that did not undergo intervention would have allowed an evaluation of mortality outcomes with conservative therapies. Lastly, although we have a robust systemwide PERT program, the overall small number of interventions may have contributed to an underpowered analysis. As experience in other programs accumulates, further evaluation in the form of registries and randomized controlled trials will be helpful in elucidating the relationship between LBT and CDT in terms of clinical outcomes. Despite these limitations, this study provides an initial glimpse into the outcomes of a head-to-head comparison of LBT and CDT.

## Conclusion

Our results suggest that LBT and CDT have similar outcomes with regard to all-cause mortality, rate of recurrent hospitalization, length of hospitalization, and bleeding outcomes. However, both these approaches have their strengths and weaknesses. At this time, there is no consensus among interventionalists with regard to the ideal method for endovascular treatment of PE. Future studies of randomized head-to-head comparison between the 2 modalities, the application of early hemodynamic support for those with high-risk PE and rapidly deteriorating hemodynamic compromise, and long-term clinical outcomes in patients undergoing invasive versus noninvasive therapies are needed.

## Figures and Tables

**Figure S1:**